# Three Cases of Post-Typhoid Neuritis

**Published:** 1896-12

**Authors:** Geo. J. Preston

**Affiliations:** Baltimore, Md.


### THREE CASES OF POST-TYPHOID NEURITIS.

By GEO. J. PRESTON, M.D.,

BALTIMORE, MD.

That neuritis is not a common complication of typhoid fever is evident from the fact that during my connection with the city hospital for several years, I have not seen a single case in the wards there. Osler reports but four cases of neuritis in 389 cases of typhoid treated at the Johns Hopkins Hospital. It is not uncommon during the course of typhoid fever to have the patient complain of pains in the limbs, and this sometimes appears to be a distinct local neuritis. Hanford and Osler have both called attention to the "tender toes," which would seem to be a mild form of local neuritis. The graver forms are apt to appear during, or shortly after, convalescence. The following are examples of the severer forms:

CASE 1. The patient, a young man, had never had any severe illness. Was taken with fever May 25th, and came to the hospital on the 30th. The fever ran a typical course, reaching 105 degrees. On July 8th he suffered a relapse, and fever again became very high. He continued in a critical condition until July 21st. With the beginning of convalescence he began to complain of pain in the legs, the slightest contact with the bed clothes producing great suffering. For three or four days there was an erysipelatous blush over the right leg, and later a small abscess developed at the ankle. The pain in the legs continued, and an examination at this time showed loss of patellar tendon reflex, some atrophy, double foot drop, and a reaction of degeneration. There was no marked disturbance of sensation. He slowly recovered the use of his limbs, and is now perfectly well.

CASE 2. The hospital nurse was taken with typhoid fever June 9th, and the disease ran a very severe course, with high temperature, and the patient was delirious for four weeks. On the fourth day after the subsidence of fever the patient complained of very severe pain in the right arm and leg, with inability to move the affected parts. In ten days the pains subsided. At this time, after spending a day in the country, the pain returned with great violence, spreading over the whole body, but most intense in the left leg. The leg showed some edema, and was hyperesthetic. At the present time there is loss of patellar tendon reflex, muscular atrophy, and the reaction of degeneration in both lower extremities, with double foot drop. The patient has very little power of motion in the lower limbs, and the grasp of the hand is very weak. There has been no disturbance of the bladder or rectum, and during the whole course of the disease very little loss of sensation.

CASE 3. Young man of 28 years developed typhoid July 25th, 1895. Previous to this he had shown signs of pulmonary tuberculosis. The disease ran a usual course of about five weeks, bed sores developed, and the temperature assumed a septic type. At this time there appeared an intense hyperesthesia most marked in the lower extremities, and paralysis of the extensor muscles of both arms and legs followed by atrophy. There was a gradual return of power, though the paralysis never entirely disappeared. The arms recovered more perfectly than the legs. The patient died from nephritis.

These cases illustrate the severer forms of neuritis, and there can be no doubt of the etiological influence of typhoid fever. A point of very great interest, and one that is not yet determined, is whether inflammation, beginning in the nerve trunk, or many nerve trunks, may, by direct continuity, involve the spinal cord. Some cases of tabies are extremely suggestive of this possibility. If such a theory can be established, then post-febrile neuritis would become of very great importance. A clinical point worth noting, and one that has attracted my attention for some years, is that cases of neuritis may present very marked motor disturbances, amounting, at times, to complete paralysis, without showing, or at least very trivial, sensory disturbances. The possibility of the etiological bearing of neuritis upon the degenerative cord diseases and the necessity of more careful treatment of the former is worthy of attention. I am a firm believer in the great utility of electricity in the treatment of neuritis, local or generative, and think that careful electrical treatment greatly hastens the recovery.

